# Mechanism of action of Taohong Siwu decoction in the alleviation of primary dysmenorrhea

**DOI:** 10.3389/fmed.2024.1343179

**Published:** 2024-04-30

**Authors:** Qixiu Zhou, Mei He, Qiong Jin, Shijia Gao, Zhuya Yang, Peifeng Zhu, Wenhong Tan, Lu Liu

**Affiliations:** Yunnan Yunzhong Institute of Nutrition and health, College of Traditional Chinese Medicine, Yunnan University of Chinese Medicine, Kunming, China

**Keywords:** Taohong Siwu decoction, primary dysmenorrhea, PI3K, AKT, signaling pathway, mechanism

## Abstract

**Background:**

As one of the most common gynecological disorders, PD significantly impacts the quality of life for women. TSD, a well-known traditional Chinese medical prescription, has gained popularity for its use in treating gynecological cold coagulation and blood stasis syndromes such as PD. However, the lack of comprehensive data hinders our understanding of its molecular mechanism.

**Purpose:**

The objective of the present study is to investigate the therapeutic effects of TSD on PD and elucidate its plausible mechanism.

**Methods:**

HPLC was employed to confirm the presence of the principal metabolites of TSD. The rat model of PD was induced by OT exposure following IWM and EB pretreatment, and subsequently treated with TSD via gastric gavage. The effects and potential mechanisms of TSD on PD rats were explored, encompassing general behavior, morphological alterations in the uterus and ovaries, biochemical indicators in the uterus and serum, and levels of proteins related to the PI3K/AKT signaling pathway.

**Results:**

Gallic acid, hydroxysafflower yellow A, albiflorin, paeoniflorin, and ferulic acid were determined to be the primary active metabolites of TSD. The pharmacological studies yielded results indicating the successful establishment of the PD model in rats. Additionally, TSD demonstrated its ability to protect PD rats by ameliorating general behavior, mitigating pathological damage to uterine and ovarian tissues, and modulating the expression levels of correlated factors (PGE2, PGF2*α*, Ca2+, TXB2, IL-6, TNF-*α*, NO, and COX-2) as well as p-PI3K/PI3K and p-AKT/AKT proteins.

**Conclusion:**

TSD exhibited protective effects against PD in rats through its interaction with multiple targets including P13K/AKT signaling pathway, indicating that TSD holds therapeutic potential for PD treatment and providing evidence supporting the rational utilization of TSD.

## Highlights

- The main metabolites TSD were identified, and were speculated to be related to its alleviation of PD.
- The protective effect and molecular mechanisms of TSD on PD were confirmed.
- TSD alleviates PD by the interaction multi-target and PI3K/AKT signaling pathway.

## 1 Introduction

Primary dysmenorrhea (PD) is a prevalent gynecological disorder observed in menstruating women, distinguished by spasmodic uterine contractions and the presence of painful symptoms linked to inflammatory disruptions, and is specifically defined as the recurring and spasmodic menstrual pain devoid of any underlying organic pathology (1–3). Although the etiology of PD has not been unambiguously elucidated, several lines of evidence suggest that excessive production and release of uterine prostaglandins (PGs) may contribute to the occurrence and progression of PD, potentially inducing abnormal uterine activity (4–6). Furthermore, it has been reported that PD may lead to elevated uterine markers, inflammatory responses, and oxidative stress, consequently impacting the levels of associated factors such as PGE2, PGF2*α*, TXB3, and Ca2+, thereby resulting in intensified and more frequent contractions and uterine ischemia (7, 8). In addition, PD has been found to be linked to sex hormones and ovarian steroid-related disorders, and its prevalence has been progressively rising in recent years, significantly affecting women’s quality of life and psychological well-being during menstruation (9–11). Therefore, the treatment of PD is utmost necessary for women, particularly during menstrual periods. Currently, nonsteroidal anti-inflammatory drugs (NSAIDs) and oral contraceptives (OCS) are commonly employed for PD treatment due to their rapid and remarkable efficacy, but they are associated with intolerable side effects on the gastroenterology, liver, kidney, gastrointestinal function, and even cardiac systems (6, 8, 12–14). Consequently, it is necessary to discover novel drug candidates that exhibit fewer side effects for the prevention and treatment of PD.

Traditional Chinese medicines (TCM) formulations such as Xiang-Fu-Si-Wu Decoction, Ge-Gen Decoction and Guizhi Fuling capsule may present a promising avenue to develop alternative and complimentary medicines for therapy and prevention of PD, owing to their peculiarity of pleiotropic action and minimal adverse reactions in the treatment of PD (15–17). As a TCM formulation with clinical efficacy in the management of menstrual disorders, Taohong Siwu Decoction (TSD), originally documented in the “The Golden Mirror of Medicine” written by Wuqian, comprised of six herbs in specific proportions, *viz. Paeonia lactiflora* Pall., *Rehmannia glutinosa* Libosch., *Angelica sinensis* (Oliv.) Diels., *Ligusticum chuanxiong* Hort., *Prunus persica* (L.) Batsch, and *Carthamus tinctorius* L. (18–20). It has been reported that TSD has been extensively utilized for the treatment of various ailments such as acute blood stasis, postpartum hemorrhage, and dysmenorrhea, due to its various pharmacological actions, including regulation of blood, antispasmodic, anti-inflammatory, and inhibition of oxidation reaction (21, 22). Despite the existing literature that has unveiled certain aspects of TSD on PD and conducted preliminary explorations of its related mechanisms, the comprehensive understanding of the relevant mechanism of TSD on PD remains incomplete, and the available data are still insufficient to substantiate the application of TSD as a treatment for PD (23). Based on the above descriptions, the current study identified 5 metabolites of TSD with High-Performance liquid chromatography (HPLC), and evaluated the curative effect of TSD on rat with PD induced by oxytocin (OT) exposure following ice-water mixture (IWM) and estradiol benzoate (EB) pretreatment, and further investigated whether TSD can regulate multi-target and PI3K/AKT signaling pathway to exert its therapeutic effect, which will contribute to a scientific testimony for elucidating the effect and mechanism of TSD in PD treatment.

## 2 Materials and methods

### 2.1 Reagents

EB and OT were provided by Hangzhou Animal Medicine Factory (Hangzhou, China). TongJingBaoKeLi (TJB, SFDA approval number: Z41021972) was bought from Zhongjing Wanxi Pharmaceutical Co., Ltd. (Henan, China). ELISA Kits for prostaglandin E2 (PGE2), prostaglandin F2*α* (PGF2*α*), thromboxane B2 (TXB2), interleukin 6 (IL-6), tumor necrosis factor-*α* (TNF-*α*), nitric oxide (NO), and cyclooxygenase-2 (COX-2) were supplied by Shanghai Enzyme-linked Biotechnology Co., Ltd. (Shanghai, China), and the Ca2+ kit was bought from Nanjing Jiancheng Bioengineering Institute (Nanjing, China). The antibodies of phosphoinositide 3-kinase (PI3K), protein kinase B (AKT), p-PI3K and p-AKT were obtained from Bioss (Beijing, China). All other reagents were analytical grade, and water was ultrapure water.

### 2.2 Preparation of TSD

Dihuang (batch No.: 21102901, origin: Henan) was provided by Yunnan Jingtian Biological Technology Co., LTD (Yunnan, China). Danggui (batch No.: 220214, origin: Gansu) and Baishao (batch No.: 220517, origin: Anhui) were purchased from Anhui Kanghe Chinese Medicine Technology Co., LTD (Anhui, China). Chuanxiong (batch No.: C220210001, origin: Sichuan) and Taoren (batch No.: C220331002, origin: Shandong) were obtained from Yunnan Zongshun Biological Technology Co., LTD (Yunnan, China). Honghua (batch No.: 20220701, origin: Yunnan) were supplied by China Resources Modern Chinese Medicine (Kunming) Co., LTD (Yunnan, China). All herbs were identified by Professor Zhuya Yang of Yunnan University of Chinese Medicine. The plant names of all the Chinese botanical drugs were checked using “The World Flora Online” (www.worldfloraonline.org).

The botanical drugs in Table 1 were mixed according to the amount, soaked in 10 times the amount of distilled water for 0.5 h, then boiled for 1 h, and filtered with double-layer gauze (200 mesh) to obtain the medicinal liquid. The extraction process was repeated twice. Finally, the obtained extracts were pooled and concentrated to dryness by lyophilization.

**Table 1:** The composition of TCM in TSD.

Chinese name	Latin name	Used part	Concentration (g herbs/500 mL decoction)
Dihuang	*R. glutinosa*	Root	11.19
Danggui	*A. sinensis*	Root	14.92
Baishao	*P. lactiflora*	Root	5.60
Chuanxiong	*L. chuanxiong*	Tuber	3.73
Taoren	*P. persica*	Seed	3.78
Honghua	*C. tinctorius*	Flower	3.73

### 2.3 HPLC analysis of TSD

The identities of the main metabolites were confirmed by HPLC analysis. Five metabolites, including gallic acid (110,831–201,906), hydroxysafflor yellow A (111,637–202,111), paeoniflorin (110,736–202,145), ferulic acid (110,773–201,915), (National Institutes for Food and Drug Control); albiflorin (PS011455), (Chengdu Pusi Biotechnology Co., LTD) were used. The separation was performed on Agilent ZORBAX SB-Aq column (250 mm × 4.6 mm, 5 μm) with acetonitrile-0.1% phosphoric acid for gradient elution, the gradient elution conditions were as follows: 0–10 min, 2% A; 10–20 min, 2% → 13% A; 20–45 min, 13% → 22% A; 45–50 min, 22% → 50% A. Flow rate was 1.0 mL/min, column temperature was 30°C. The detection wavelength was set at 225 nm (0 ~ 35 min) and 280 nm (35 ~ 50 min).

### 2.4 Animals

Female SD rats (6–8 weeks), SPF, were purchased from Hunan SJA Laboratory Animal Co., Ltd. (license number: SCXK (Xiang) 2019–0004). All animals, were routinely housed in the Centre of Laboratory Animal Administration of the Yunnan University of Chinese Medicine, had free access to food and water and were exposed to a 12 h light/dark cycle for one week before experimentations. The temperature was 23 ± 2°C, and the relative humidity was 60 ± 20% in this environment.

### 2.5 Preparation and treatment of animal models

Throughout the experiment, the hair, vitality, and weight of all rats were observed and documented daily. Sixty rats were randomly divided into the following 6 groups (n = 10 per group) after 1 week of adaptive feeding: the normal control group (NC); the model control group (MC); the positive control group (PC: TJB, 2.1 g/kg); the low-dose TSD group (TSD-L: 4.5 g/kg); the medium-dose TSD group (TSD-M: 9.0 g/kg); and the high-dose TSD group (TSD-H: 13.5 g/kg). The PD model of rats was established according to previous research (24). Except for rats in the NC group, rats in the other groups received intragastric administration of EB (0.4 mg/kg, the first and last day doses were double) once daily for 12 consecutive days, and the rats were couple-stimulated using an IWM (0–4°C) administered to the lower abdomen once daily (20 min per time) to establish the model of PD with CCBS. On day 5 of modeling, the rats in treatment groups were treated by gavage with different doses of TSD and TJB once daily for 7 consecutive days, while the rats in the NC and MC groups were intragastrically administered with equivalent distilled water. On day 12 of modeling, rats in the NC group were intrauterinely injected with normal saline after the last injection of EB for 1 h, but rats in the other groups were intrauterinely injected with OT (2 IU/rat).

### 2.6 Behavioral observation of rats

After that, the latency period and frequency of writhing response in rats for 30 min were monitored and recorded to evaluate the PD model, and explore the effect of TSD on pain in rats of this model.

### 2.7 Collection of biological samples

After the behavioral observation of rats, the blood samples were extracted from abdominal aorta after anesthetizing rats with chloral hydrate and centrifuged at 3500 rpm and 4°C for 15 min to obtain serum, and then serum were collected into EP tubes. The rats were subsequently sacrificed that their uterus and ovaries were isolated and weighed, and the viscera indicators were calculated (viscera index (%) = organ mass (mg)/body mass (g) × 100%). And the uterus and ovaries on the left side of rats in each group were fixed with 4% paraformaldehyde, and the remaining uterus and ovaries were stored at −80°C like serum for further use.

### 2.8 Histopathological examinations

Hematoxylin–eosin (H&E) staining was performed based on the standard protocol of manufacturer. Briefly, paraffin tissue sections of uterus and ovaries were dewaxed and dehydrated using a decreasing concentration gradient, followed by staining with hematoxylin and eosin, and then sealed with neutral resin. Subsequently, morphological characteristics were photographed through a light microscope following a randomized selection of the viewing field.

### 2.9 Biochemical analysis

The contents of PGE2, PGF2*α*, Ca2+, and TXB2 in uterine tissues of rats, as well as the levels of IL-6, TNF-*α*, NO, and COX-2 in the serum of rats, were measured using kits in accordance with the instructions of the manufacturer.

### 2.10 Western blotting

To explore the underlying mechanism of the action of TSD on PD, WB was used for determining the levels of proteins such as PI3K, AKT, p-PI3K, and p-AKT in uterine tissues. The tissues were lysed with RIPA Lysis Buffer for 30 min in an ice bath to obtain total proteins, and then their concentrations were determined using a BCA protein assay kit. After subjecting with 15% SDS-polyacrylamide gel electrophoresis, the proteins were transferred to a polypropylene fluoride (PVDF) membrane, and blocked at room temperature for 2 h, followed by overnight incubation at 4°C with primary antibodies for the aforementioned proteins. Subsequently, secondary antibodies were added at room temperature for 2 h. Protein bands were obtained and analyzed based on the gray value.

### 2.11 Statistical analysis

All the experimental data were presented as means ± standard deviation (SD) of three independent analyses. Statistical analysis was carried out using one-way ANOVA (GraphPad Prism 8.0.2). Differences at *p* values <0.05 were considered to be statistical significance. Results were processed using the computer programs Excel software.

## 3 Results

### 3.1 Analysis of the chemical metabolites of TSD by HPLC

**Figure 1:**
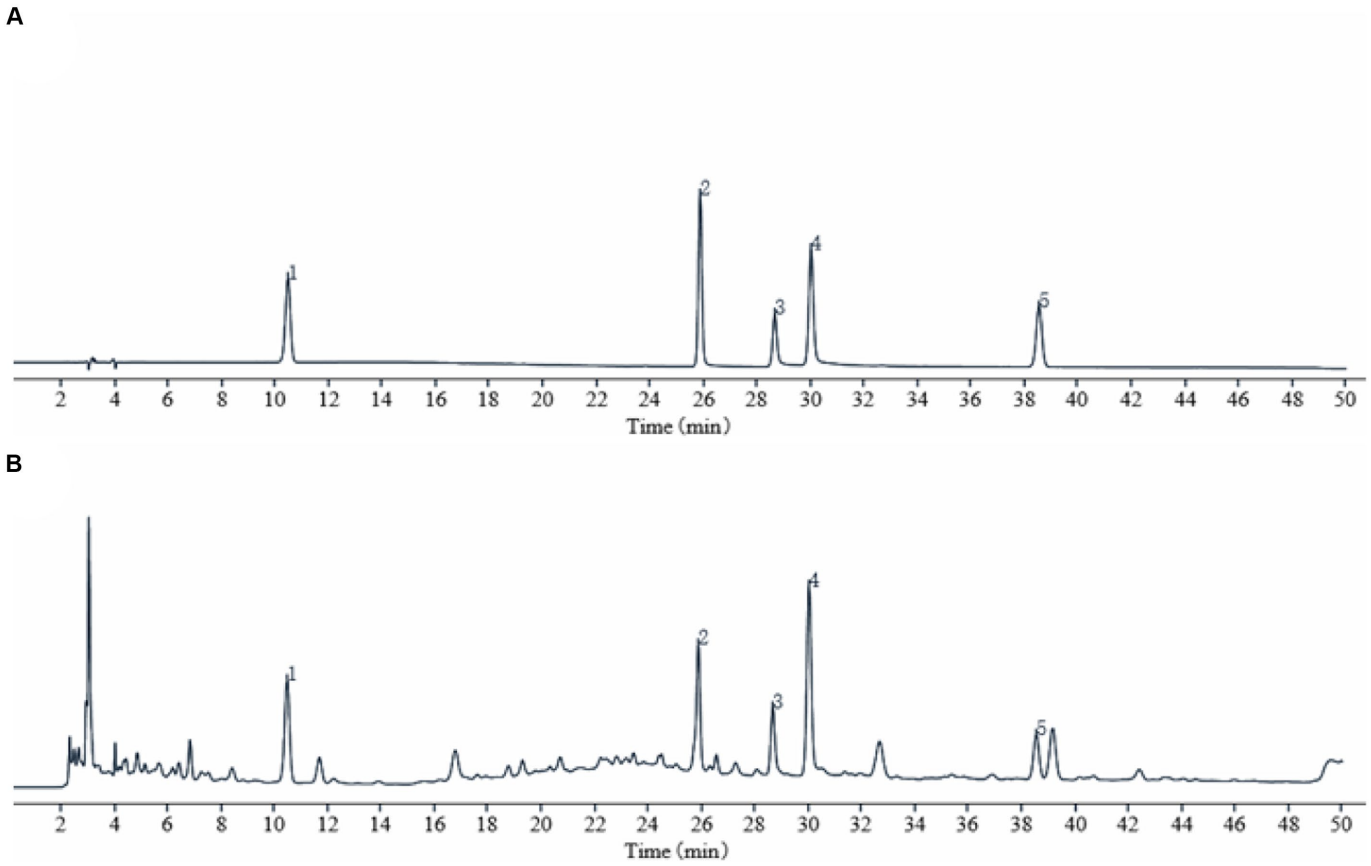
HPLC chromatogram of TSD. **(A)** Standard product; **(B)** TSD. Identification of main metabolites in TSD as following: gallic acid (1), hydroxysafflower yellow A (2), albiflorin (3), paeoniflorin (4), and ferulic acid (5).

The chromatograms of the mixed standards and TSD were shown in Figure 1. In this study, the prominent chromatographic peaks in TSD were qualitatively analyzed by the retention time of the standard, which showed that the retention times of gallic acid, hydroxysafflower yellow A, albiflorin, paeoniflorin, and ferulic acid were at 10.45, 25.85, 28.64, 30.00, and 38.52 min, respectively. Subsequently, the above metabolites were quantitatively analyzed by an external standard method, and then calculated by the regression curve, which showed that their mass fractions in TSD were 0.54, 2.40, 2.40, 3.91, and 0.56 mg/g, separately. These results indicated that the above five metabolites were the main metabolites of TSD.

### 3.2 Effect of TSD on pathological signs of model rats

#### 3.2.1 Behavioral analysis of rats

**Figure 2:**
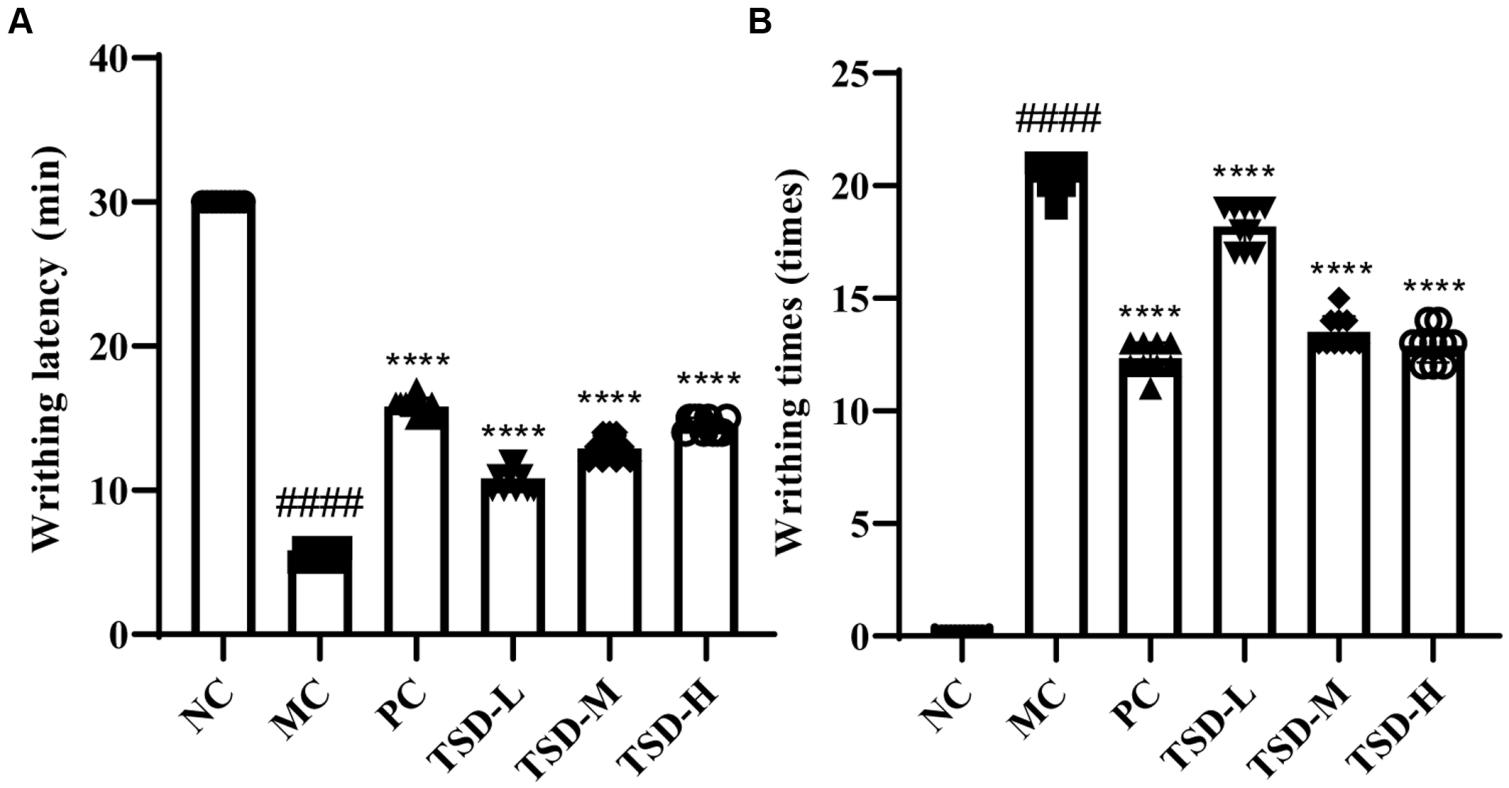
Effects of TSD on the writhing of rats. **(A)** Writhing latency; **(B)** writhing times. ^####^*p* < 0.0001 versus the NC group; **p < 0.01, ***p < 0.001 and ****p < 0.0001 versus the MC group.

As descripted in Figure 2, compared to the rats in the NC group, weight loss, hair removal, and irritable mood were observed in the modeling process amongst the MC group rats with significantly reduced latent period before writhing and significantly frequent twisting times. After TJB and TSD administration, gradually recovered weight, hair softness and slightly stable mood were observed in rats with significantly prolonged writhing latency and significantly decreased twisting times, as compared to the rats in the MC group. The results showed that the PD model was successfully established using EB, IWM, and OT, while the abnormal behaviors of rats with PD were alleviated by TSD.

#### 3.2.2 The viscera index

**Figure 3:**
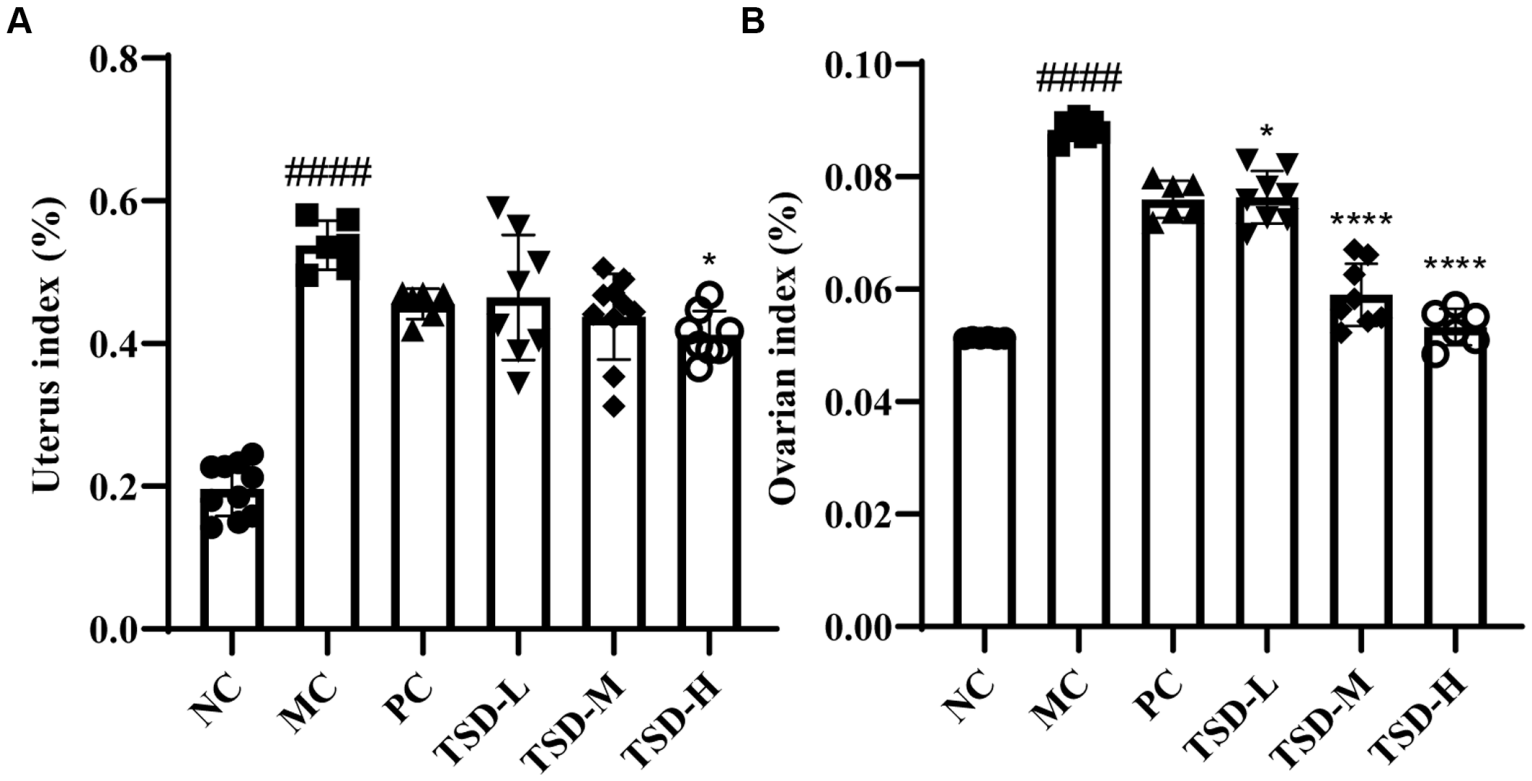
The viscera indexes of rats. **(A)** Uterus index; **(B)** ovarian index. ^####^*p* < 0.0001 versus the NC group; *p < 0.05 and ****p < 0.0001 versus the MC group.

The conditions of uterus and ovaries for rats were presented by viscera index, as indicated in Figure 3. Compared with rats in the NC group, uterine and ovarian indexes in rats in the MC group were significantly increased. After drug administration, elevated uterine and ovarian indexes in rats were relieved in the treatment groups, especially in the TSD groups, and uterine and ovarian indexes decreased with increased TSD dose, as compared to rats in the MC group. These findings suggested that the uterus and ovaries were enlarged and damaged in rats with PD, which could be treated by TSD.

#### 3.2.3 Pathological manifestations of uterus and ovaries

**Figure 4:**
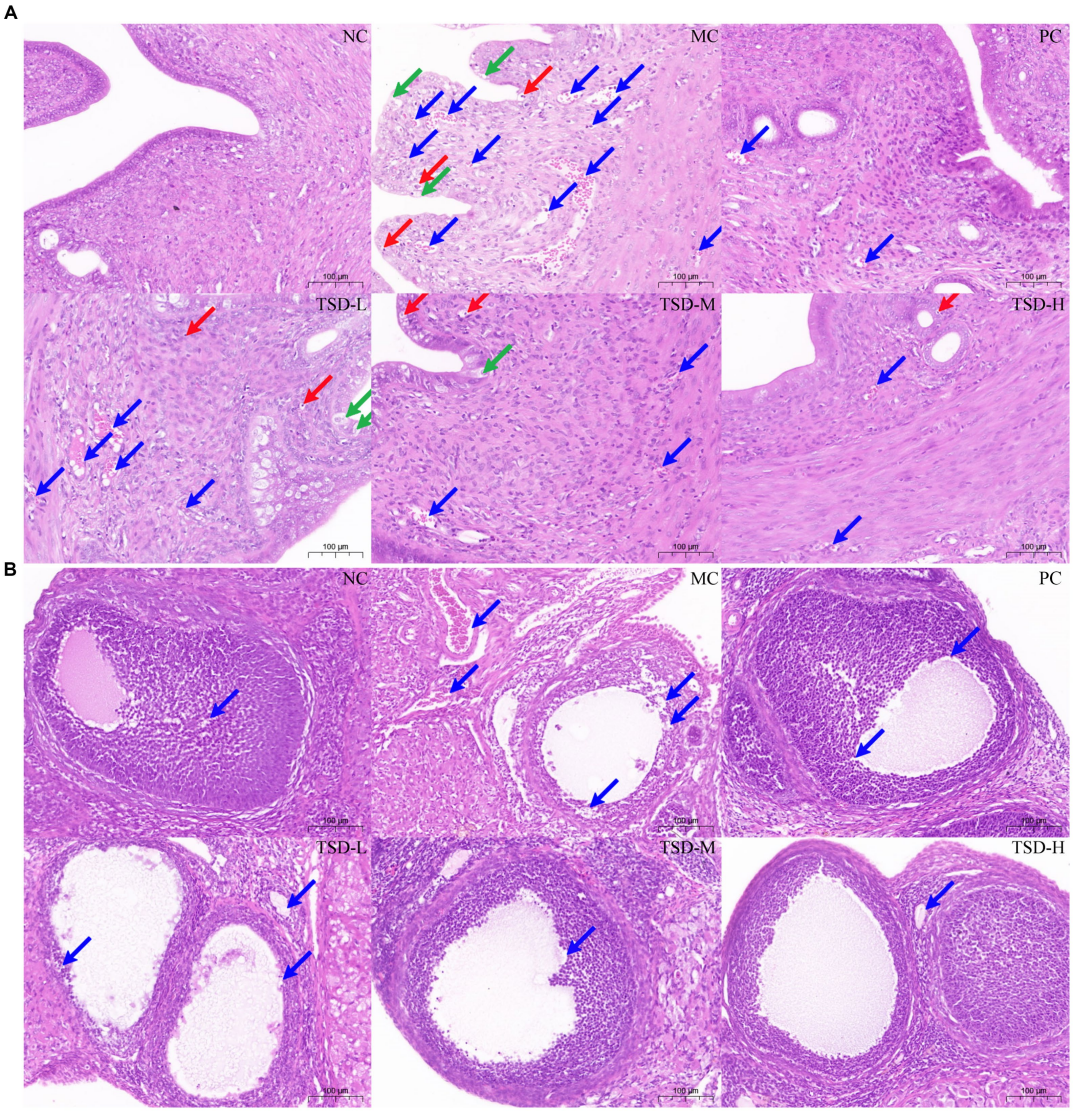
The results of H&E staining on uterine and ovarian tissues in rats (100×). **(A)** The histopathology examination of uterus: Red arrows represent punctate necrosis of cells or nucleus fragmentation; Blue arrows represent granulocyte and lymphocyte infiltration; Green arrows represent the inside membrane epithelium vacuole degeneration and cytosolic vacuoleization. **(B)** The histopathology examination of ovaries: Arrows represent nucleus fragmentation, cytoplasm relaxation, and reduction or disappearance of the follicular granular layer.

The pathological manifestations of the uterus and ovaries were analyzed and recorded, as shown in Figure 4A, there were no obvious abnormalities in the uterine tissue of rats from the NC group. However, the uterine tissue of rats in the MC group showed that the endometrial epithelium has punctate necrosis, nucleus fragmentation, cytosolic vacuolization, granulocytes and lymphocytes were infiltrated, and the cell arrangement was disordered. After the intervention of TJB and TSD, the above pathological changes were obviously alleviated to varying degrees. Inflammatory infiltration was reduced, and cellular organization exhibited relatively normal characteristics.

In addition, there were no obvious abnormalities in the ovarian tissue of rats from the NC group (Figure 4B). However, these phenomena occur in the ovarian tissue of rats in the MC group, including nucleus fragmentation, cytoplasm relaxation, and reduction or disappearance of the follicular granular layer. After TJB and TSD intervention, nucleus fragmentation and cytosoloporosis were improved, the follicle granular layer was relatively intact, and ovarian morphology became clear. In summary, rats in the MC group exhibited pathological damage to uterine and ovarian tissue, whereas this damage was ameliorated by TSD intervention, which confirmed that the successful establishment of PD models in rats and demonstrates the efficacy of TSD on PD.

### 3.3 Effect of TSD on biochemical indicators in uterus and serum of PD rats

**Figure 5:**
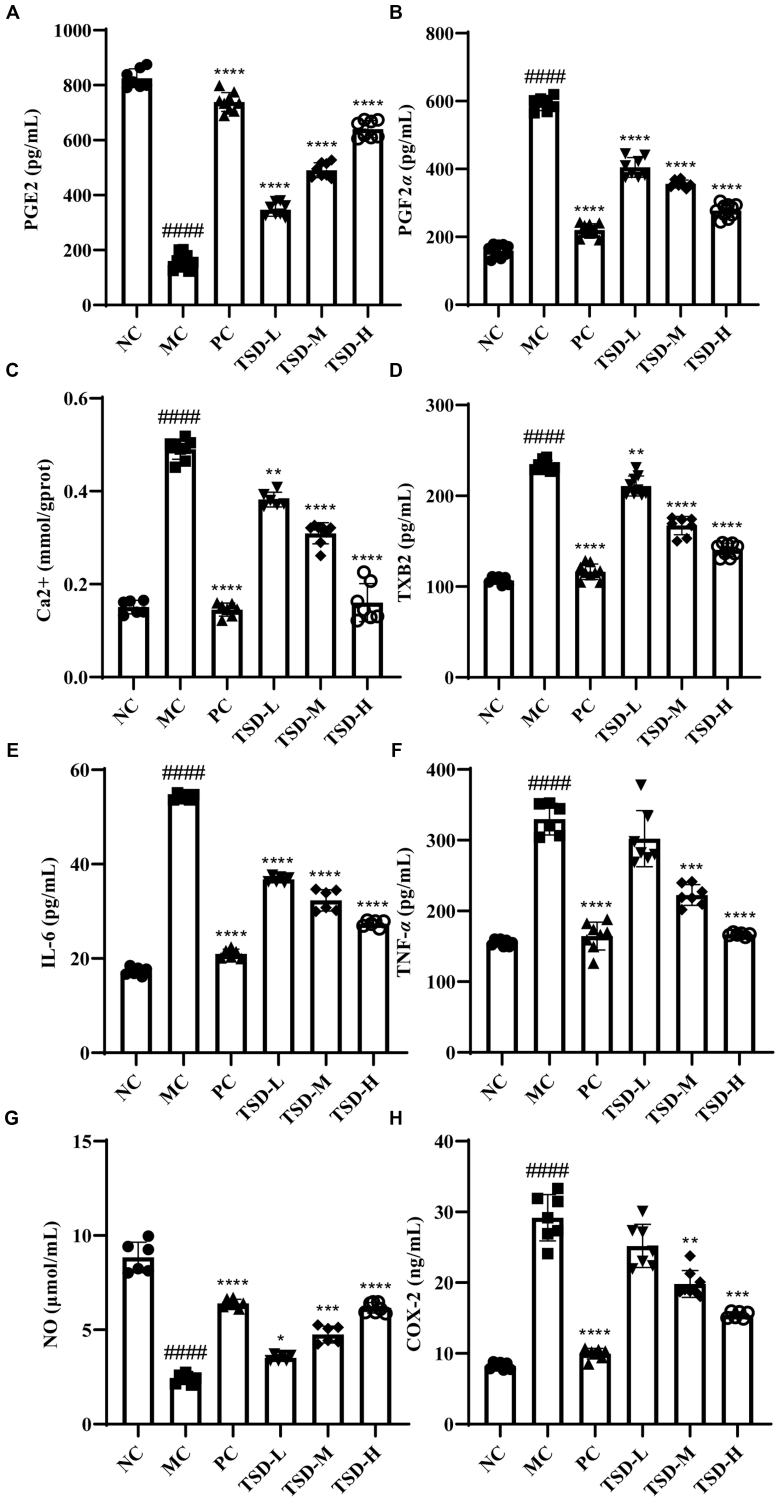
Effects of TSD on biochemical factors in rats with PD. **(A)** the level of PGE2; **(B)** the level of PGF2*α*; **(C)** the level of Ca2+; **(D)** the level of TXB2; **(E)** the level of IL-6; **(F)** the level of TNF-*α*; **(G)** the level of NO; **(H)** the level of COX-2. ^####^*p* < 0.0001 versus the NC group; *p < 0.05, **p < 0.01, ***p < 0.001 and ****p < 0.0001 versus the MC group.

As illustrated in Figure 5, compared with those of the rats in the NC group, the contents of PGE2 and NO in uterine tissues of rats from the MC group were significantly reduced, while the levels of PGF2*α*, Ca2+, TXB2, IL-6, TNF-*α*, NO, and COX-2 in serum of rats from the MC group were significantly elevated a conversely changing trend, revealing that the rats were successfully induced to establish PD model. After treatment with drugs, compared with the MC group, the contents of PGE2 and NO in uterine tissues were significantly increased, but the levels of PGF2*α*, Ca2+, TXB2, IL-6, TNF-*α*, and COX-2 in serum were significantly decreased, indicating that TSD could treat PD rats and its mechanism may be closely related to the regulation of the above indicators.

### 3.4 Effect of TSD on the expression of related proteins in uterus of PD rats

**Figure 6:**
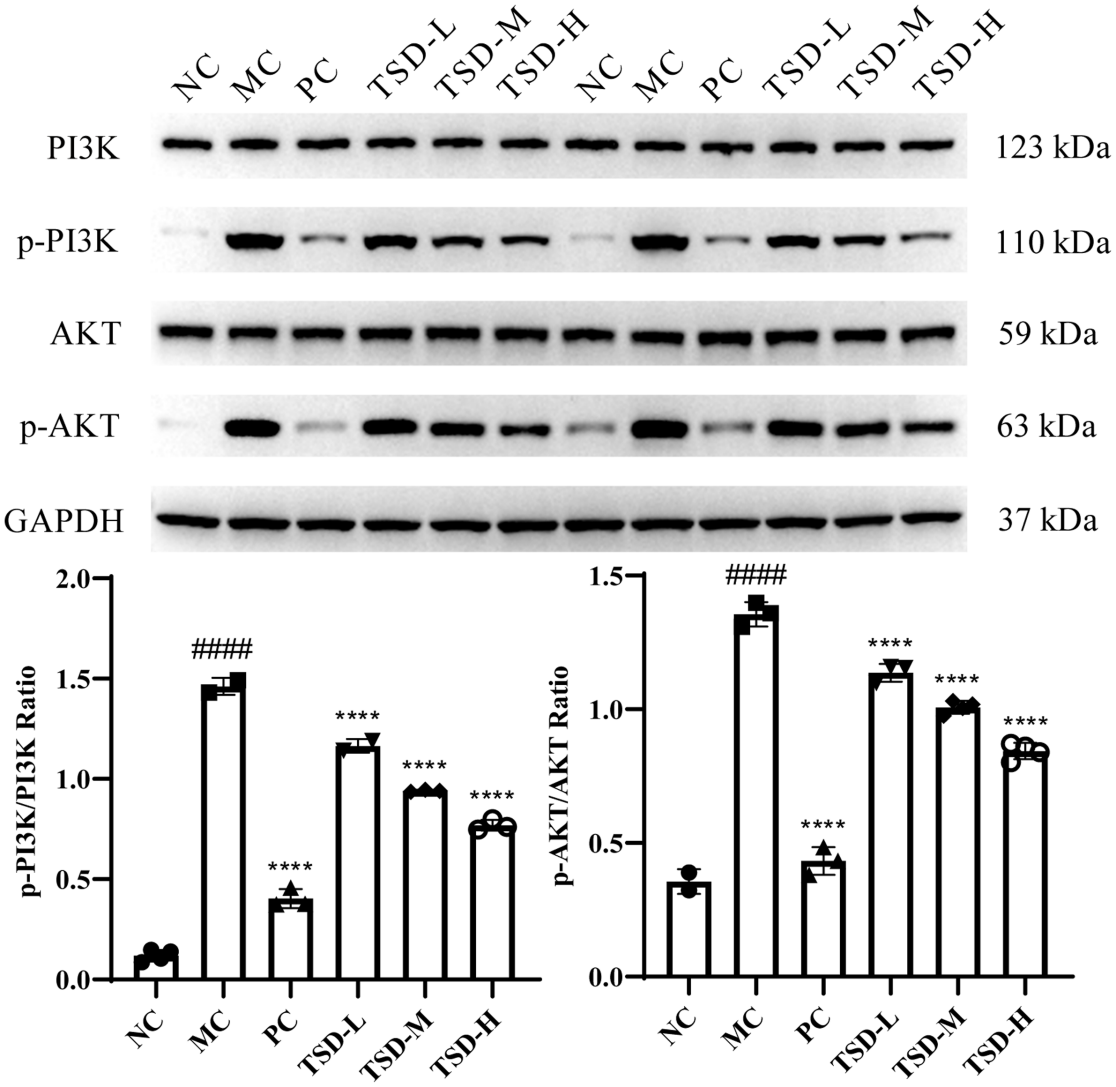
The expression of PI3K, AKT, p-PI3K, and p-AKT in the uterine tissues of rats. Each band was presented as a representative figure, and a histogram was calculated from the band density value of at replicate and independent experiments. GAPDH was used as an internal control. ^####^*p* < 0.0001 versus the NC group; ****p < 0.0001 versus the MC group.

The protein levels of PI3K, AKT, p-PI3K, and p-AKT in the uterus of rats were described in Figure 6. The levels of PI3K and AKT were generally consistent in MC group compared to NC group, while the p-PI3K and p-AKT levels were significantly upregulated. After drugs administration, PI3K and AKT levels were generally consistent compared with the MC group, whereas the p-PI3K and p-AKT levels were significantly downregulated, and surprisingly, the p-PI3K and p-AKT levels were dose-dependent decreased in the TSD groups. Therefore, the ratios of p-PI3K/PI3K and p-AKT/AKT were obviously increased in the MC group when compared to the NC group. However, compared to the MC group, the p-PI3K/PI3K and p-AKT/AKT ratios were significantly reduced after drugs administration. The results suggested that TSD may affect the above protein expression levels in the PI3K/AKT signaling pathway, thereby protecting PD rats.

## 4 Discussion

The prevalence and severity of gynecological disorders, particularly PD, have significantly increased, negatively impacting women’s quality of life. And with the vigorous development of TCM, which has emerged as a crucial therapeutic approach in managing various diseases. TSD, a renowned TCM prescription, has been extensively utilized for treating gynecological conditions associated with blood stasis, including PD. However, the underlying molecular mechanisms of TSD’s therapeutic effects remain poorly elucidated. Therefore, this study aims to investigate the protective effect of TSD treatment on PD and explore the associated mechanisms, potentially involving inhibition of certain pathways.

In this study, the HPLC chromatographic peaks of TSD were identified with standards, and it was confirmed that the known metabolites were gallic acid, hydroxysafflower yellow A, albiflorin, paeoniflorin and ferulic acid in TSD. Hydroxysafflower yellow A, paeoniflorin, and ferulic acid are the index metabolites of Honghua, Baisao, Danggui and Chuanxiong for the quantitative detection in the Chinese pharmacopoeia 2020. Gallic acid exists in a variety of Chinese botanical drugs, but the content is high in TSD, and it can reduce the levels of inflammatory factors such as NO, IL-6, and PGE2, and regulate the activities of prostatin synthase and thromboxane synthase, thereby controlling the production of TXA2 and prostacyclin (15, 25, 26). Hydroxysafflower yellow A can improve the sluggish of poor circulation and hemorrheology parameters of rats with blood stasis syndrome (27). Albiflorin has been widely used to treat pain and inflammation (28). Studies have shown that paeoniflorin is active against dysmenorrhea within a certain dose range, and ferulic acid can improve uterine blood supply and relieve dysmenorrhea (29, 30). Therefore, it is speculated that they may be the main active metabolites in the treatment of PD with TSD.

EB can promote estrus in female rats, OT can induce excessive uterine contraction, IWM can provide cold coagulation conditions, and pathological signs of rats can be used to evaluate model establishment (15, 17, 31). Therefore, in this study, the rat PD model was replicated and evaluated by EB, IWM and OT, indicating that the rat PD model was effectively replicated. Then, the PD rats were treated with TSD, which explained that TSD could improve the general behavior, twisting response, uterine and ovarian indexes, and pathological damage to uterine and ovarian tissue in PD rats.

PGF2*α* and TXA2 are PGs with biological activities produced by the human uterus. PGF2*α* can cause uterine contractions, leading to the accumulation of anaerobic metabolites that stimulate pain receptors, and the concentration of PGF2*α* increases with the onset of PD (32, 33). TXA2 is one of the most effective endogenous factors involved in platelet stimulation with short half-life and unstable characteristics, its abnormal fluctuations can lead to Ca2+ influx, induce platelet aggregation, vasospasm, among others, and activated Ca2+ can regulate the production of inflammatory cytokines, so abnormal changes of TXA2 may lead to PD (34, 35). However, TXB2 is a relatively stable active metabolite produced by TXA2 during metabolism, so it is commonly used to evaluate the content of TXA2, which is used as a marker of PG *in utero* (36–38). IL-6 and TNF-*α* can induce inflammatory responses, stimulate the production of prostaglandins from arachidonic acid and accelerate the contraction of uterine smooth muscle (39, 40). It has been reported that the mechanism of albiflorin alleviating dysmenorrhea may be closely related to an increase in NO levels and a decrease in COX-2 levels in uterine tissue (41). Therefore, the levels of PGE2, PGF2*α*, Ca2+, TXB2, IL-6, TNF-*α*, NO, and COX-2 were examined to evaluate the therapeutic effect of TSD on PD, which showed that the abnormal changes of these indicators could be improved by TSD, indicating that TSD has a good therapeutic effect on PD rats, and its mechanism may be closely related to the regulation of the above indicators.

The P13K/AKT pathway has elaborate mechanisms or pathways. P13Ks and AKT are involved in a variety of mechanisms including cell survival, wherein P13K signaling cascade can regulate many essential functions for cell survival (42). As a canonical downstream signaling effector of PI3K, AKT, which is also known as protein kinase B, regulate a number of important cellular processes such as cell growth, proliferation, survival, migration, invasion, tissue invasion and angiogenesis (43). Furthermore, the PI3K/AKT signaling pathway plays a crucial role in the regulation of platelet function that it can induce platelet granule release, platelet activation and platelet adhesion (44). PI3Ks are a family of intracellular signal transducer enzymes, and play an extremely important role in platelet functional responses. The isoform of PI3Ks, PI3Kβ has been confirmed by pharmacological and genetic studies to be extremely important for the regulation of platelet function (33, 45). In addition, the restoration of PI3K/AKT signaling pathway can reduce apoptosis of cells (46). Therefore, in this study, the PI3K/AKT signaling pathway was used as the foothold to further explore the mechanism of TSD in the treatment of PD. The results displayed that the expression levels of p-PI3K/PI3K and p-AKT/AKT in rats with PD were significantly reduced by TSD, demonstrating that TSD may modulate the PI3K/AKT pathway by inhibiting the expression levels of related proteins to maintain cell survival, regulate platelet function and suppress cell apoptosis in uterine and ovarian tissues, thereby protecting the ovaries and uterus to achieve the purpose of treating PD.

## 5 Conclusion

In this study, the main metabolites of TSD, including gallic acid, hydroxysafflower yellow A, albiflorin, paeoniflorin and ferulic acid, were speculated to be related to its alleviation of PD, and the treatment effect and trend of TSD in PD treatment via modulation of multi-target and PI3K/AKT signaling pathway were confirmed. However, it is noteworthy that drug treatment for diseases typically involves other enriched metabolites and pathways, and the PI3K/AKT signaling pathway has a very elaborate mechanisms or pathways for regulation. Therefore, further research is warranted to systematically characterize the molecular mechanism of TSD in the treatment of PD, which provides a more favorable insight for the rational utilization of TSD to treat PD in the future.

## Data availability statement

The datasets presented in this study can be found in online repositories. The names of the repository/repositories and accession number(s) can be found in the article/Supplementary material.

## Ethics statement

The animal study was approved by the Ethics Committee of Yunnan University of Chinese Medicine. The study was conducted in accordance with the local legislation and institutional requirements.

## Author contributions

QZ: Conceptualization, Data curation, Formal analysis, Investigation, Methodology, Software, Visualization, Writing – original draft, Writing – review & editing. MH: Conceptualization, Data curation, Formal analysis, Investigation, Methodology, Software, Visualization, Writing – original draft, Writing – review & editing. QJ: Data curation, Formal analysis, Methodology, Supervision, Validation, Writing – review & editing. SG: Data curation, Formal analysis, Methodology, Supervision, Validation, Writing – review & editing. ZY: Data curation, Formal analysis, Methodology, Supervision, Validation, Writing – review & editing. PZ: Data curation, Formal analysis, Methodology, Supervision, Validation, Writing – review & editing. WT: Conceptualization, Funding acquisition, Investigation, Methodology, Project administration, Resources, Supervision, Writing – review & editing. LL: Conceptualization, Funding acquisition, Investigation, Methodology, Project administration, Resources, Supervision, Writing – review & editing.
